# Aortic coarctation with anomalous aortic origin of left coronary arteries

**DOI:** 10.1093/ehjcr/ytag278

**Published:** 2026-04-21

**Authors:** Shi-Qin Yu, Yuan Li, Zhi-Gang Yang

**Affiliations:** Department of Radiology, West China Hospital, Sichuan University, 37# Guo Xue Xiang, Chengdu, Sichuan 610041, China; Department of Radiology, West China Hospital, Sichuan University, 37# Guo Xue Xiang, Chengdu, Sichuan 610041, China; Department of Radiology, West China Hospital, Sichuan University, 37# Guo Xue Xiang, Chengdu, Sichuan 610041, China

## Case description

**Figure 1:**
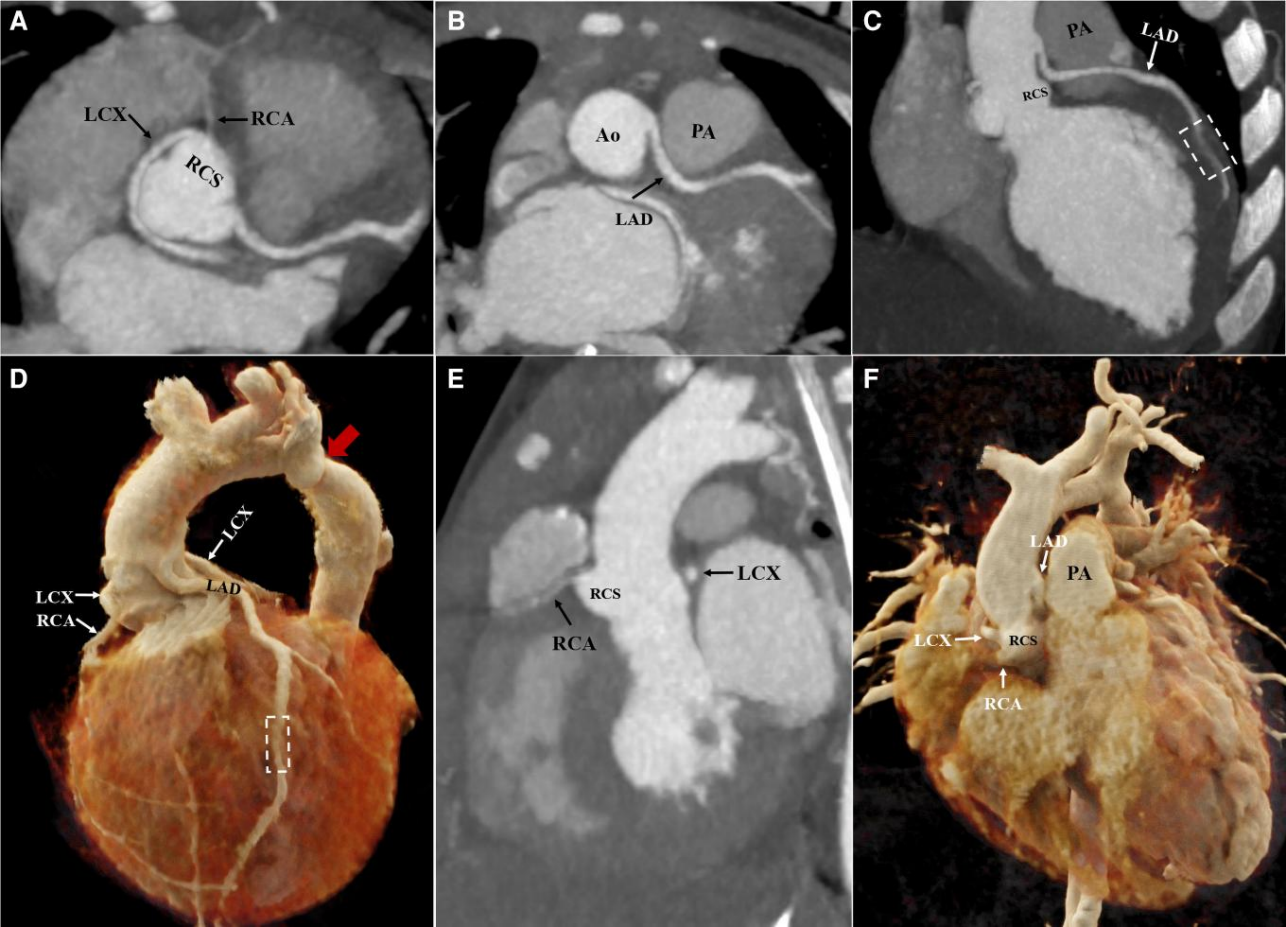
Computed tomography angiography images from a 5-month-old male infant with aortic coarctation and coronary artery anomalies. (*A–C* and *E*) Maximum intensity projection images. (*A*) Both the left circumflex artery and right coronary artery originated from the right coronary sinus. (*B* and *C*) The left anterior descending artery originated 4 mm above sinotubular junction of the right coronary sinus with an interarterial course and demonstrated a myocardial bridge in its mid-segment (dashed box). (*D* and *F*) Cinematic rendering showed coarctation of descending aorta (red arrow) along with the anomalous origin and course of coronary arteries. Ao, aorta; PA, pulmonary artery.

A 5-month-old male infant presented with a cough following a clod 1 week prior, which rapidly progressed to feeding difficulties and dyspnoea. Initial evaluation at a referring hospital identified aortic coarctation with a 55 mmHg pressure gradient, severe mitral regurgitation, and reduced left ventricular systolic function on echocardiography, alongside severe pneumonia on chest computed tomography (CT). Due to progressive hypoxaemia, the patient required invasive mechanical ventilation and was transferred to our institution for surgery. The electrocardiogram showed no evidence of myocardial ischaemia or arrhythmias. Pre-operative cardiac CT angiography incidentally revealed multiple coronary anomalies: (i) the left circumflex artery originated from the right coronary sinus (RCS) via a separate ostium with a retroaortic course (*Figure 1A*); (ii) the left anterior descending artery originated 4 mm superior to the sinotubular junction of the RCS with an interarterial course (*Figure 1B* and *C*) and exhibited a 7 mm long myocardial bridge in the mid-segment (*Figure 1C* and *D*, dashed box); (iii) the right coronary artery was hypoplastic and arose from the RCS (*Figure 1A* and *D–F*). The coarctation of the descending aorta demonstrated a minimum diameter of 3.4 mm (*Figure 1D*, red arrow). Given the patient’s young age and the absence of obvious compression of the anomalous left coronary arteries, coarctation resection with end-to-end anastomosis was performed via lateral thoracotomy. At the 1-month follow-up, echocardiography showed normalized left ventricular ejection fraction.

While the combination of aortic coarctation and anomalous origin of coronary artery from pulmonary artery has been well-documented,^1^ the coexistence of anomalous aortic origin of left coronary arteries with aortic coarctation is a rare finding.^2^ Features in this condition, including interarterial course and orifice anomalies (slit-like orifice, acute take-off angle and high take-off), are considered high-risk anatomy that can lead to myocardial ischaemia and sudden cardiac death.^3^ Optimal management remains debated as the exact pathophysiology of life-threatening events is incompletely understood. Management strategies should be tailored to individual patient characteristics, including age, symptoms, evidence of ischaemia, and compression or narrowing of the ectopic artery.

## Data Availability

No new data were generated or analysed in support of this research.
